# Effect of LASER therapy Vs conventional techniques on clinical and radiographic outcomes of deciduous molar pulpotomy: A systematic review and meta-analysis

**DOI:** 10.4317/jced.56436

**Published:** 2020-06-01

**Authors:** Vennila Chandran, Venkitachalam Ramanarayanan, Medhini Menon, Balagopal Varma, Vinita Sanjeevan

**Affiliations:** 1 Department of Pedodontics and Preventive Dentistry, Amrita School of Dentistry, Amrita Vishwa Vidyapeetham, Kochi, Kerala, India; 2 Department of Public Health Dentistry, Amrita School of Dentistry, Amrita Vishwa Vidyapeetham, Kochi, Kerala, India; 3 Department of Public Health Dentistry, Goa Dental College, Bambolim, Goa, India

## Abstract

**Background:**

To systematically review the effectiveness primary molar pulpotomy based on the clinical and radiographic outcomes using lasers over the conventional therapies.

**Material and Methods:**

This systematic review and meta-analysis included Randomized or Quasi-randomized trials comparing LASER with conventional pulpotomy therapies (formocresol, ferric sulphate, MTA or calcium hydroxide) with atleast 6-month follow-up period was included. Risk of bias of included studies was assessed and metanalysis was done using RevMan software.

**Results:**

Of the 1383 articles that were searched, only 14 studies were included for qualitative synthesis and 10 for meta- analysis. There was no statistically significant difference in clinical success rate [OR 0.99, 95%CI (0.19,5.22)] or radiographic success rate [OR 0.77, 95%CI (0.31,1.87)] of LASER therapy compared to Formocresol in primary molar pulpotomy for 6 months. No statistically significant difference were found in clinical success rate [OR 1.04, 95%CI (0.35,3.07)] and radiographic success rate [OR 0.71, 95%CI (0.37,1.35)] at 12 month follow-up also. Comparison of LASER with Ferric Sulphate also did not show a statistically significant difference.

**Conclusions:**

Meta-analysis showed no statistically significant difference in clinical and radiographic outcomes of LASER pulpotomy with conventional pulpotomy (formocresol and ferric sulphate) at 6 and 12 months follow-up. However, there was considerable risk of bias in the included studies.

** Key words:**Pulp therapy, Laser, formocresol.

## Introduction

Dental pulp can be defined as a connective tissue of mesodermal origin, which is highly vascularized and innervated structure enclosed by dentin with communications to the periodontal ligament. The pulp present in the crown and root are called coronal pulp and radicular pulp respectively. A vital pulp is essential to good dentition. The preservation of a vital pulp during operative procedures and successful management in case of disease are the most important challenges to the clinical dentist. Vital pulp therapy plays a major role in the preservation of primary dentition after caries affliction (1).

Currently, there are three vital pulp therapy (VPT) options for treatment of deep dentin caries lesions approximating the pulp in vital primary teeth viz. indirect pulp cap, direct pulp cap (DPC) and pulpotomy (2). The pulp is an organ with remarkable reparative abilities (3). Pulpotomy is the most preferred pulp therapy when only the coronal pulp is inflamed due to bacterial penetration following carious, traumatic or iatrogenic causes, and the radicular pulp is free from inflammation (4). and it still continues to be the most prevalent vital pulp therapy.

According to Finn (1995), pulpotomy is defined as the complete removal of the coronal portion of the dental pulp, followed by placement of a suiTable dressing or medicament that will promote healing and protects vitality of the pulp. The remaining vital radicular pulpal tissue were treated with different pulpotomy medicaments through three different mechanisms viz. devitalization (such as formocresol, electrosurgery, and LASERtherapy), preservation (such as ferric sulfate and glutaraldehyde) and regeneration (such as MTA, calcium hydroxide) (5).

Since 1950, formocresol (FC) has been the widely used material for pulpotomy of primary molars (6). A successful pulpotomy depends on various important factors like case selection, clinical diagnosis, intraoperative diagnosis and most importantly the material used for the pulpotomy procedure. Formocresol pulpotomy (FC) is still a popular pulp therapy despite the concerns raised due to its toxicity, mutagenicity and carcinogenicity due to a good clinical and radiographic success rates (7).

Ferric Sulfate (FS) a non-aldehyde chemical has gained attention recently as a pulpotomy agent. This haemostatic agent was proposed on the theory that it prevents the problem in clot formation thereby reducing the chances of inflammation and internal resorption (7).

Mineral Trioxide Aggregate (MTA) is a newer material used for pulpotomies with a high rate of success. Clinical trials supported that MTA performs equal to or better than formocresol or ferric sulfate and may be the most desirable pulpotomy agent in the future (8).

The electrosurgical pulpotomy(ES) and the LASER pulpotomy, are the forms of non chemical devitalization that emerged during the last decade. The electrocautery carbonizes and denatures the pulp and prevents bacterial contamination (9).

A recently emerged non pharmacotherapeutic method, was the use of LASER, in which the LASER energy is able to overcome the histologic deficits of electrosurgery by accelerating the wound healing of the pulp and also the expression of the lectins and collagen levels. The advantage of LASER has also been suggested as an alternative, owing to its hemostatic, antimicrobial, and cell-stimulating properties with minimal alteration to the pulpal tissue (10). It is therefore considered as a complementary step to the pulpotomy process in primary teeth (11). One of the benefits of using LASER in pediatric dentistry is its ability for selective and precise interaction with diseased tissues. Only a minimal thermal necrosis of adjacent tissues is produced with LASERS than with electrosurgical instruments (12).

Based on the review of literature it has been found that the primary teeth can be treated with many different techniques and medicaments, but the success rates of these newer pulpotomy medicaments are still uncertain due to the varying results about the clinical, radiographical and histopathological outcomes.

Hence the objective/PICO statement of this study is to perform a systematic review and meta-analyses, to evaluate the effectiveness primary molar pulpotomy (Population) based on the clinical and radiographic outcomes (Outcomes) using LASERS (Intervention) over the conventional therapy, amongst the pulpotomy medicaments like Formocresol, MTA, Ferric sulfate and Calcium hydroxide (Control).

## Material and Methods

-Protocol and Registration

The protocol of the study was registered with Prospero (International Prospective Register of Systematic Reviews) with Register no: CRD42018093035.

-Eligibility criteria

Randomized or Quasi-randomized trials comparing two or more pulpotomy techniques of which one was LASER and the other was any of the conventional therapies (formocresol, ferric sulphate, MTA or calcium hydroxide) with atleast 6-month follow-up period was included.

-Types of participants

Children (4 to 10 years of age) undergoing pulpotomy therapy in vital primary molars with carious pulp exposure using LASER treatment and formocresol and/or ferric sulphate and/or mineral trioxide aggregate and/or calcium hydroxide were the study participants. Children with any systemic disorders or pulpotomy conducted on teeth other than primary molars were excluded from the study.

Intervention group consisted LASER therapy for primary molar pulpotomy and comparator groups were Conventional techniques for primary molar pulpotomy (formocresol, ferric sulphate, MTA and biodentine).

-Information sources and search

The literature search was done with the electronic databases viz. The Cochrane Oral Health’s Trials Register; The Cochrane Central Register of Controlled Trials (CENTRAL) (The Cochrane Library); MEDLINE Ovid (from 1946 onwards); Google Scholar; ProQuest and EBSCO. Search was also done based on references of included studies and hand searching was also attempted.

No limitations were imposed on the date and country of the publication, but only trials published in the English language were included in the review. The search strategies for databases were modelled on that designed for MEDLINE Ovid.

Key words used to identify relevant studies were (“LASERS” AND (“MTA” OR “formocresol” OR “ferric sulphate” OR “biodentine”) AND “pulpotomy”); (“LASERS” AND “MTA” AND “pulpotomy”); (“LASERS” AND “formocresol” AND “pulpotomy”); (LASER) AND pulpotomy; (“LASERS” AND “ferric sulphate” AND “pulpotomy”); (“LASERS” AND “biodentine” AND “pulpotomy”).

-Study selection

The screening of titles and the abstracts of potentially relevant articles was performed by VC and RV before retrieving full articles. The risk of bias using the recommendations in the Cochrane Handbook for Systematic Reviews of Interventions has been individually assessed by VC and RV independently. The disagreements if any, were to be resolved by discussion or, if necessary, by consulting a third review author (MM) in order to reach a consensus.

-Data items

A data extraction form was prepared in Microsoft Excel by RV. Both the authors (VC and RV) agreed on the design of the data extraction form. In every included trial, characteristics of the participants, details of the interventions applied and outcomes were entered. VC and RV independently extracted the data and were not blinded to the authors of the included studies.

-Outcome measures

Primary outcome measures were assessment of clinical and radiographic success separately after 6 months and 12 months of procedure.

-Assessment of risk of bias

We evaluated all the studies, which were included for analysis, by the recommended approach for assessing the risk of bias (13). All the included studies were assessed independently and in duplicate by two review authors (VC and RV) for study design characteristics and features of internal validity. Assessment was done within and across studies. We assessed for publication bias as recommended in Cochrane Handbook using funnel plots.

-Summary measures

The treatment effect was summarized into dichotomous outcomes using odds ratio.A method of fixed-effects model were used to calculate a pooled estimate of effect and its 95% confidence intervals (CIs).

-Synthesis of results and Additional analyses

Meta-analysis was performed for methodologically similar studies. We concluded the heterogeneity based on patient demographics, clinical circumstances, and the comparability of the interventions applied. We also evaluated of the heterogeneity of the data using Cochran’s Q statistic, a Chi2 test, with a threshold *p* value of less than 0.10. The consistency of the results was assessed visually using forest plots and by the I2 statistic (14).

Meta-analysis could be performed only for LASER Vs Formocresol and LASER Vs Ferric sulphate as sufficient studies were not available for other included interventions (MTA, biodentine and calcium hydroxide).

## Results

**Figure 1:**
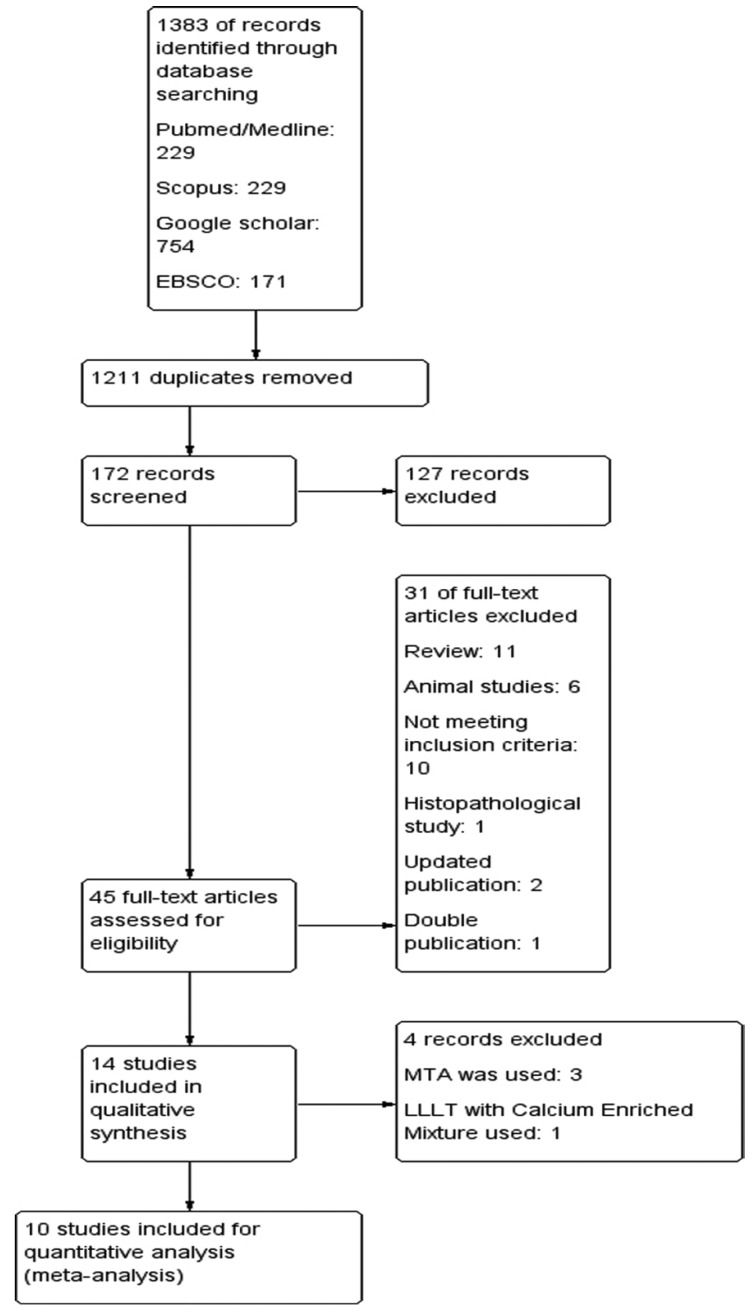
Search strategy and PRISMA flow diagram.

About 14 studies were included for the qualitative synthesis while 10 studies were included for quantitative synthesis (meta-analysis). The PRISMA flow diagram is given in Figure 1. All the included studies were Randomized Controlled Trials and sample size ranged from 20 to 200. The studies were conducted from 2005 to 2018. Since the follow-up times were varied across studies, it was decided to include 6 months and 12 months follow up clinical and radiographic values for this study. Study participants were children age 3 to 9 years and study setting were mostly dental clinics. The characteristics of included studies are given in Table 1.

**Table 1:**
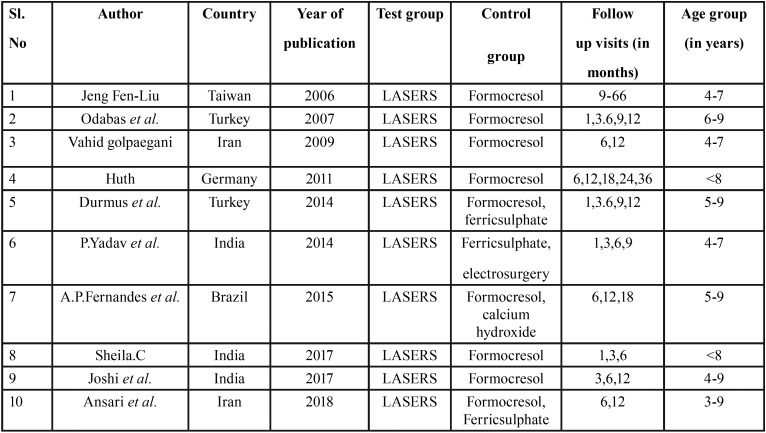
Characteristics of included studies.

Risk of bias within studies and across studies

Table 2 and  Table 3 show the risk of bias across the studies and within studies respectively. The assessment of risk of bias was indicating a high risk of the included studies. No study included in the study was free of all the biases. All studies except that of Joshi *et al.* did not blind the participants. Allocation concealment was also not attempted in most of the studies. However, there was low risk of bias as far as attrition of study participants and selective reporting was concerned.

**Table 2:**
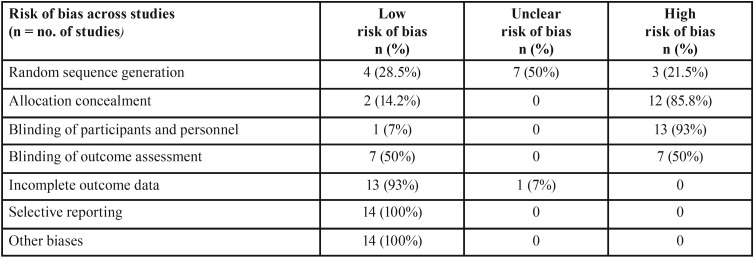
Risk of bias across studies.

**Table 3:**
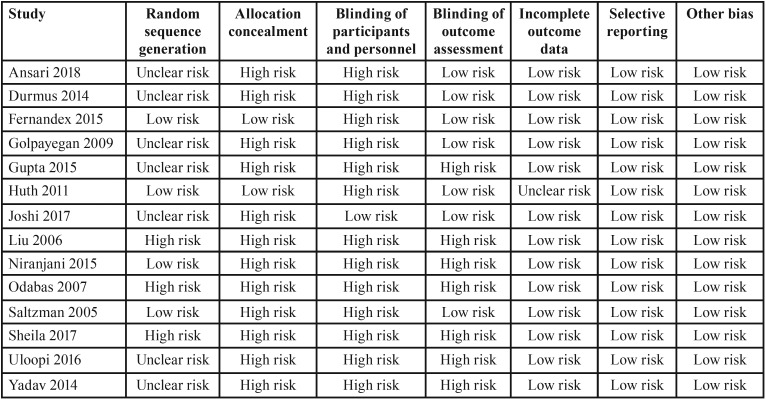
Risk of bias within studies.

Effect of interventions

-LASER pulpotomy Vs Formocresol

•Clinical and Radiographic success after 6 months

**Figure 2:**
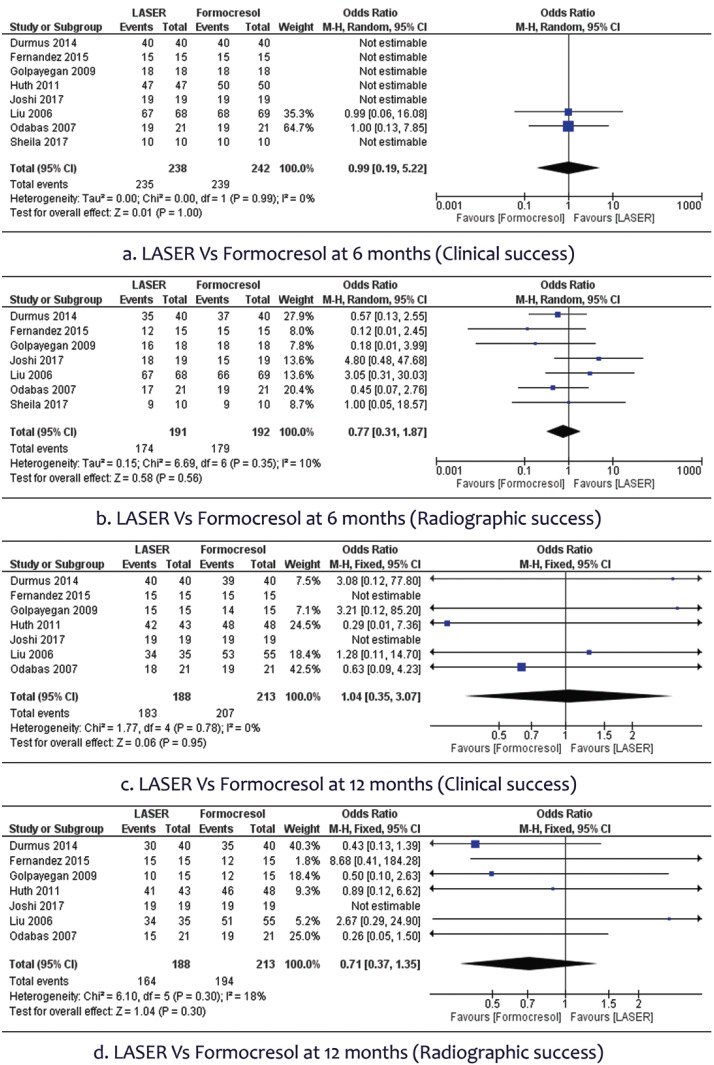
Forest plot of Laser Vs Formocresol.

In 8 studies involving the 480 participants, we found no statistically significant difference in clinical success rate [OR 0.99, 95% CI (0.19 to 5.22)] of LASER therapy when compared to the conventional Formocresol therapy in primary molar pulpotomy (Fig. 2a). A total of 7 studies with 383 participants assessed the radiographic success rate, which did not show any statistically significant difference between the two groups [OR 0.77, 95% CI (0.31 to 1.87)]. There was no heterogeneity (0%) across studies (Fig. 2b).

•Clinical and Radiographic success after 12 months

In 7 studies involving the 401 participants, no statistically significant difference were found in clinical success rate [OR 1.04, 95% CI (0.35 to 3.07)] of LASER therapy when compared to the conventional Formocresol therapy in deciduous molar pulpotomy after 12 months (Fig. 2c). A total of 7 studies with 401 participants were available to assess the radiographic success rate and found no statistically significant difference between the two groups [OR 0.71, 95% CI (0.37 to 1.35)]. There was slight heterogeneity (<3%) across studies (Fig. 2d).

-LASER pulpotomy Vs Ferric sulphate

•Clinical and Radiographic success after 6 months

**Figure 3:**
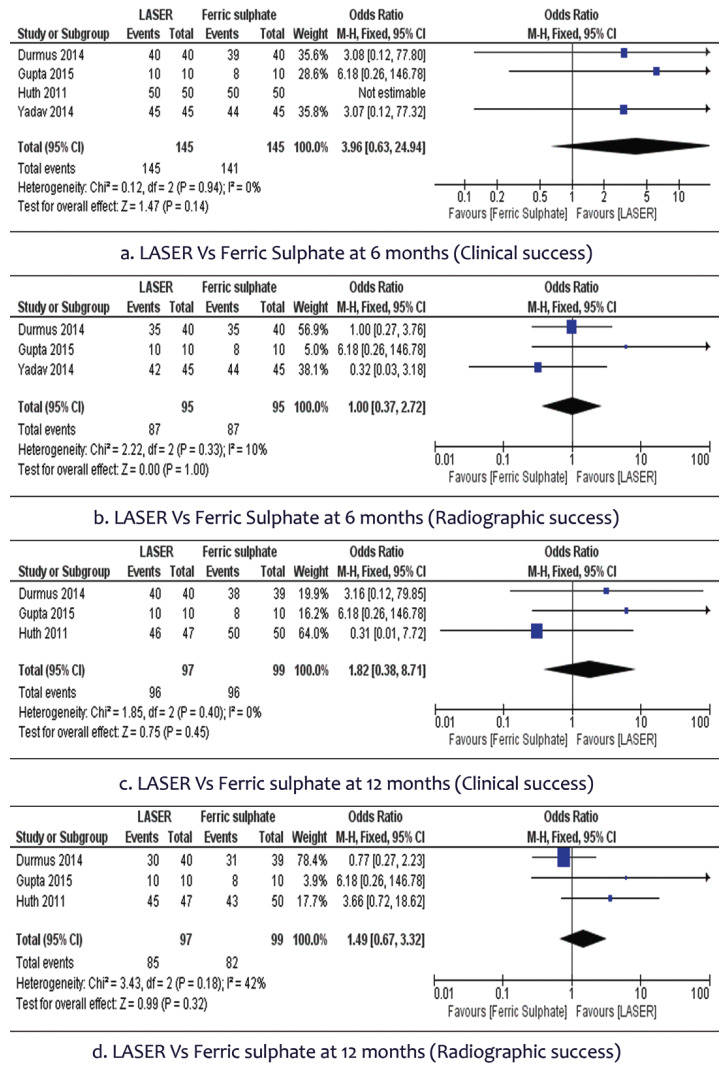
Forest plot of Laser Vs Ferric sulphate.

In 4 studies involving the 290 participants, we found no statistically significant difference in clinical success rate [OR 3.96, 95% CI (0.63 to 24.94)] of LASER therapy when compared to the conventional Ferric sulphate therapy in primary molar pulpotomy. There was no heterogeneity (0%) across studies (Fig. 3a). A total of 3 studies with 190 participants assessed the radiographic success rate and did not show any statistically significant difference between the two groups [OR 1, 95% CI (0.37 to 2.72)]. There was slight heterogeneity (10%) across studies (Fig. 3b).

•Clinical and Radiographic success after 12 months

In 3 studies involving the 196 participants, no statistically significant difference were found in clinical success rate [OR 1.82, 95% CI (0.38 to 8.71)] of LASER therapy when compared to the conventional Ferric sulphate therapy. There was no heterogeneity (0%) across studies (Fig. 3c). A total of 3 studies with 196 participants were available to assess the radiographic success rate and found no statistically significant difference between the two groups [OR 1.49, 95% CI (0.67 to 3.32)]. There was moderate heterogeneity (42%) across studies (Fig. 3d).

A symmetrical funnel plot was obtained for all outcomes having more than 5 studies.

## Discussion

One of the ultimate goals for all pediatric dentists is to preserve the primary dentition in good health until it succumbs to normal exfoliation. These teeth are vital for proper mastication and speech, maintenance of arch length, and are crucial for the overall health of children. Early childhood caries is one of the major reasons for early pulpal involvement of primary teeth and unfortunately the prevalence of ECC estimated in a study in 2012 has shown to be in between a range of 50%- 80% (15). A recent study by Anil S *et al.* in 2017 has shown a soaring prevalence of 85% in developing countries and in the underprivileged population in the developed countries (16).

Taking into consideration this alarming prevalence, pulpotomy serves as one such vital pulp therapy that comes into rescue to conserve these primary teeth. In pulpotomy only coronal pulp is removed with a rationale that the radicular pulp may be healthy or is capable of self healing.

Pediatric dental practitioners are still in a constant hunt for the most ideal medicament to be used in pulpotomy. Among the numerous factors that decide the success or failure of pulpotomy, the medicament/technique used forms the touchstone of pulpotomy (17). The ideal medicament for pulpotomy should possess the following prerequisites: non toxicity, bactericidal, biocompatible to pulp and surrounding structures, aid in healing of the remaining radicular pulp without impeding physiologic root resorption (18).

Formocresol has been the gold standard and has been in use for the last 70 years and is still extensively used among pedodontists due to its ease of application and high clinical success rates (19). Nevertheless many studies have shown cytotoxicity and mutagenecity to be its hazardous effects (20). Thereafter various medicaments like gluteraldehyde, calcium hydroxide, ferric sulfate, freeze dried bone and newer materials like MTA and biodentine were introduced into pulpotomy and innumerable studies have been conducted comparing the success of each of these medicaments. Recently non pharmacological methods like electrosurgery and LASERS have also been used extensively for pulpotomy.

Use of ferric sulfate has been studied widely and it is shown to act like a hemostatic agent during pulpotomy. A metalloprotein complex is formed, when ferric sulfate contacts with blood, and the membrane of this ferric ion protein seals and block vessels mechanically, producing hemostasis (21). This agglutinated protein plugs occludes the capillary orifices preventing irritating components of subbase to enter the radicular pulp (22). It showed enhanced action in minimizing inflammation and internal resoprtion when compared to calcium hydroxide as it aided in physiologic clot formation. The material has various advantages like easy availability, no heat is produced, no electricity required and good handling properties (23). LASERS possess absorption effect on tissues that lead to cellular vaporization and ablation, that aid in proper hemostasis. This vaporization effect causes molecular bond breaking and photobiochemical disruption that causes destruction of bacteria. LASERS help in healing by expression of lectins and collagens (17). This systematic review analyzed all published clinical trials that compares LASER with conventional pulpotomy medicaments mainly formocresol and ferric sulfate.

LASER Versus Formocresol

The study conducted by Mesut Enes Odabas *et al.* (2007) compared the clinical, radiographic and histopathologic outcomes of formocresol pulpotomy and ND:YAG LASER pulpotomy in 18 teeth planned for serial extraction with a following period of 1,3,6,9 and 12 months. This study was one among very few studies that has compared the histopathologic findings post LASER and formocresol pulpotomy. The clinical, radiographic and histopathologic success rates was found to be not statistically significant till the 12th month follow up, but there was a statistically significant difference between the 7th day and 60th day follow up within the LASER group, when considering the inflammatory cell response criteria by Hebling *et al.* There was moderate inflammatory cell infiltration on 7th day but had reduced to only slight infiltration around the exposure site (24). Sheila C *et al.* in 2017 conducted a randomized, single blind split mouth study of 20 teeth to compare the clinical and radiographic success rates of Formocresol versus diode LASER pulpotomy. Both clinically and radiographically, the results were insignificant at the 6th and 12th month follow up (17). A study by Vahid *et al.* compared Low level LASER therapy pulpotomy to conventional Formocresol pulpotomy both clinically and radiographically at 6 and 12 months. At the end of 6th and 12th month both groups showed statistically insignificant results (25). According to Utsunomiya *et al.* LLLT accelerated wound healing and expression of lectins and collagens in study done on dental pulp of dogs (26). Ferreira *et al.* showed that LLLt induces a positive effect on the reactional dentinogenesis in human teeth (27). Another study by Ana Paula *et al.* in 2015 also assessed the effectiveness of LLLT on 60 vital deciduous molars and compared it both clinically and radiographically with formocresol as well as calcium hydroxide pulpotomy. There was a fourth group which was included where LLLT preceded placement of calcium hydroxide. After 6 months, the RSR was 100% for FC group, 60% for CH group, 80% and 87.5% for LLLT and LLLt +Ch group respectively. At 12 months, FC remained same whereas CH group reduced to 50% and other two were 80% and 78.6% respectively (28).

Contradictory to the above mentioned studies two studies showed that LASER was better than conventional material used for pulpotomy and only one among those studies showed statistically significant results. Liu JF *et al.* in 2006 compared Nd YAG LASER with formocresol for pulpotomy in deciduous molars. This was a long follow up study and the 68 teeth that were included in LASER group and 69 teeth in formocresol group were followed up from 6 – 68 months revealead that the success rate of Nd YAG LASER both clinically and radiographically were significantly higher than that of formocresol pulpotomy.(29) Another similar study by Elliot *et al.* comparing CO2 LASER and FC pulpotomy on healthy non carious primary canines which statistically insignificant difference among two groups (30). Liu JF *et al.* stated that this could be because study done by Elliot *et al*. included non carious teeth with smaller sample size (n=15) and a shorter follow period of 3 months (versus 68 months) (29).

The second study was a recent study conducted by Joshi *et al.* in 2017, on 40 teeth (2 in each child) that required pulpotomy. The teeth were randomly allocated to either diode LASER or formocresol group. All cases were followed up at 3, 6, 12 months. Although radiographically LASER fared much better than formocresol, the results among two groups were statistically insignificant. Failures in the formocresol group was because only the clot separated the eugenol from the vital tissue, hence direct contact from the sub base which is zinc oxide eugenol lead to its hydrolysis, releasing free eugenol. Free eugenol induces inflammatory tissue response. The cause for failures in LASER group suggested by Joshi *et al.* are carbonization, necrosis and infiltration of inflammation cells, edema in pulp tissue as a sequelae of LASER irradiation which could have caused the pathology (31).

LASER Versus Ferric Sulfate 

P Yadav *et al.* in 2014 compared the effects of ferric sulfate, electrosurgical and diode LASER pulpotomy on 45 human deciduous molars (n=15) at 1,3,6,9months. The overall CSR of FS group was 86.6%, whereas diode LASER and ES group both showed 100% CSR. Radiographically, also the results were not statistically significant (23). A similar study comparing the same three modalities in pulpotomy of primary molars was conducted by Garima *et al.* in 2015. A total of 10 teeth were allotted to each group and followed up till 12 months. The results were not statistically significant both clinically and radiograhically (9).

Ansari G *et al.* compared formocresol, ferric sulfate, calcium enriched mixture with and without LLLT pulpotomy. Formocresol showed highest clinical success rate at 12 months but they concluded that LLLT may give favorable outcomes due to faster pulp healing. Ferric sulphate had more failures the reason the authors suggest is the difference in mechanism of action, that is formocresol causes fixation whereas ferric sulphate can only help in hemostasis (11). Fernandez CC *et al.* in their study in 2013 stated that higher number of radiographic failures were in FS group, as this material only caused hemostasis which has higher chance of inflammation in longer terms unlike formocresol that causes fixation of underlying tissue (32). Huth *et al*. in 2012 assessed the long term (36 months) effectiveness of FC, FS, CH AND LASER and found that although not statistically significant LASER was as good as placing formocresol (33).

From the above studies mentioned in this study, it is evident that LASERS fare as good a pulpotomy medicament as the gold standard formocresol and ferric sulphate and although it requires trained operator skill. The advantages of LASERS while working on children are reduced chair side time, painless procedure and that high speed airotors are not required.

## Conclusions

Although the results of this study when comparing LASER v/s formocresol and LASER v/s ferric sulphate, both the clinical and radiographical follow up at 6 and 12 months, shows that the difference is not statistically significant, the studies included carried high risk of bias.

## Clinical relevance

• Pulpotomy technique is a time tested and is the most preferred procedure in primary teeth with deep carious lesions (with probable pulp exposure) amongst pediatric dentists.

• The results of this study show that the success rates of LASER pulpotomy versus pulpotomy with other medicaments are very similar and difference among the two are not statistically significant, showing that LASERS serve as an equally good alternative to other conventional pulpotomy medicaments escpecially formocresol which is considered as the gold standard for pulpotomy. LASER is also a safer option than formocresol pulpotomy, taking into consideration the reported cytotoxic and mutagenic properties of formocresol.

• This evidence based data would benefit all pediatric dentists and general dental practitioners in choosing an appropriate technique for pulpotomy.
